# Impact of hyperlipidemia and atrial fibrillation on the efficacy of endovascular treatment for acute ischemic stroke: a meta-analysis

**DOI:** 10.18632/oncotarget.20183

**Published:** 2017-08-11

**Authors:** Jingwei Zheng, Ligen Shi, Weilin Xu, Ningning Zhao, Feng Liang, Jingyi Zhou, Jianmin Zhang

**Affiliations:** ^1^ Department of Neurosurgery, Second Affiliated Hospital, School of Medicine, Zhejiang University, Hangzhou, Zhejiang, China; ^2^ Brain Research Institute, Zhejiang University, Hangzhou, Zhejiang, China; ^3^ Collaborative Innovation Center for Brain Science, Zhejiang University, Hangzhou, Zhejiang, China; ^4^ Department of Endocrinology, The Children's Hospital, School of Medicine, Zhejiang University, Hangzhou, Zhejiang, China

**Keywords:** endovascular treatment, acute ischemic stroke, meta-analysis, hyperlipidemia, atrial fibrillation

## Abstract

**Introduction:**

Stroke is the crucial cause of death annually. Inconsistent results from the randomized controlled trials (RCTs) aroused controversy on efficacy of endovascular treatment (EVT).

**Materials and Methods:**

To evaluate the efficacy of EVT in stroke patients. We searched three databases including PubMed, Web of science and the Cochrane Library from Jan 2011 to Apr 2017. Eligible studies were RCTs comparing EVT versus standard medical therapy alone. The primary outcomes were favorable functional outcomes (modified Rankin Scale score, 0–2) at 3 months. Meta regression analysis and subgroup analysis were used to explore potential influence factors responsible for the effectiveness of EVT.

**Results:**

Eleven RCTs involving 3018 patients were included in our study. EVT showed better functional outcomes at 90 days (OR, 1.71; 95% CI, 1.28–2.28; *P* < 0.001) and a higher recanalization rate at 24h (OR, 6.49; 95% CI, 4.79–8.79; *P* < 0.001). In meta-regression and subgroup analysis, primary outcomes were significantly better among patients with atrial fibrillation (Adj R-squared 46.30%, *P* = 0.054; OR, 2.40; 95% CI, 1.81–3.19; *P* < 0.001), patients without hyperlipidemia (Adj R-squared 35.21%, *P* = 0.159; OR, 2.34; 95% CI, 1.80–3.04; *P* < 0.001) and when new generation thrombectomy device was used (Adj R-squared 72.21%, *P* = 0.011; OR, 2.14; 95% CI, 1.75–2.61; *P* < 0.001).

**Conclusions:**

EVT showed superior clinical outcomes compared with standard medical therapy. The rate of using new generation thrombectomy device was the critical factor influencing therapeutic outcome. Hyperlipidemia and atrial fibrillation may also cause the potential effect.

## INTRODUCTION

Stroke is thought to be the main cause of death and disability-adjusted life-years lost globally [1]. Especially in recent years, we are confronted with the severe consequences caused by stroke [1, 2]. The first proven medical treatment of acute ischemic stroke (AIS) is intravenous thrombolysis with tissue plasminogen activator (t-PA) [3]. The recanalization rate of intravenous thrombolysis is not ideal in major large vessel occlusion [4]. Moreover, only limited patients are eligible for the therapy of t-PA because of the transient therapeutic time window (< 4.5 hours) [5] and other strict qualified criteria for administration (a history of intracranial hemorrhage, coagulation abnormalities, recent surgery) [6, 7].

In order to improve the recanalization rate, endovascular treatment (EVT) which includes mechanical thrombectomy and intra-arterial t-PA, has appeared as the alternative treatment for several years [8]. First generation thrombectomy techniques involve a thrombus aspiration by coil retrievers. With the improvement of endovascular techniques, the second generation device (Solitaire flow restoration device) could be placed within the thrombus and extract the clot as the stent is withdrawn [9]. Compared with medical therapy alone, mechanical thrombectomy can significantly increase the recanalization rate of large artery occlusion with Solitaire flow restoration device (Solitaire FR) [10, 11]. This is thought to be the crucial factor of favorable functional outcomes in ischemic stroke patients [12]. In theory, the use of mechanical thrombectomy combined with intravenous t-PA should express their respective advantages including favorable recanalization rate and quick administration [13]. However, the functional outcomes of four randomized control trials (IMS III [14], MR RESCUE [15], SYNTHESIS [8], THERAPY [16]) showed no significant difference between EVT and medical therapy. It may be attributed to the reasons that intra-arterial t-PA or first-generation mechanical thrombectomy devices played a primary role in EVT, patients were enrolled without imaging evidence of large vessel occlusion and the irregular use of IV t-PA in EVT group. On the contrary, EVT showed its overwhelming advantages in both favorable functional outcomes and recanalization rate in RCTs after 2015 (THRACE [13], EXTEND-IA [17], MR CLEAN [18], SWIFT PRIME [19], REVASCAT [20], ESCAPE [21], PISTE [22]). These RCTs improved the above-mentioned limitations by enrolling patients with the confirmation of large vessel occlusion, regular use of IV t-PA, and a higher rate of using thrombectomy devices in EVT group. The different results of diverse RCTs have aroused great interest of researchers. Continuous RCTs and meta-analysis were performed to assess the effectiveness of EVT and the relative influence factors [4, 23, 24].

Previous meta-analysis used pre-specified subgroups to find out the possible reasons of heterogeneity in functional outcomes between different therapies. This approach may lead to the neglect of potential important factors [4, 23, 24]. Hence, we pooled data from eleven eligible multicenter RCTs to explore the efficacy and safety of EVT for AIS. Simultaneously, a mathematical method would be implemented to observe all the possible factors that might affect therapy outcome without undue man-made interference.

## RESULTS

### Search results and study characteristics

**Figure 1:**
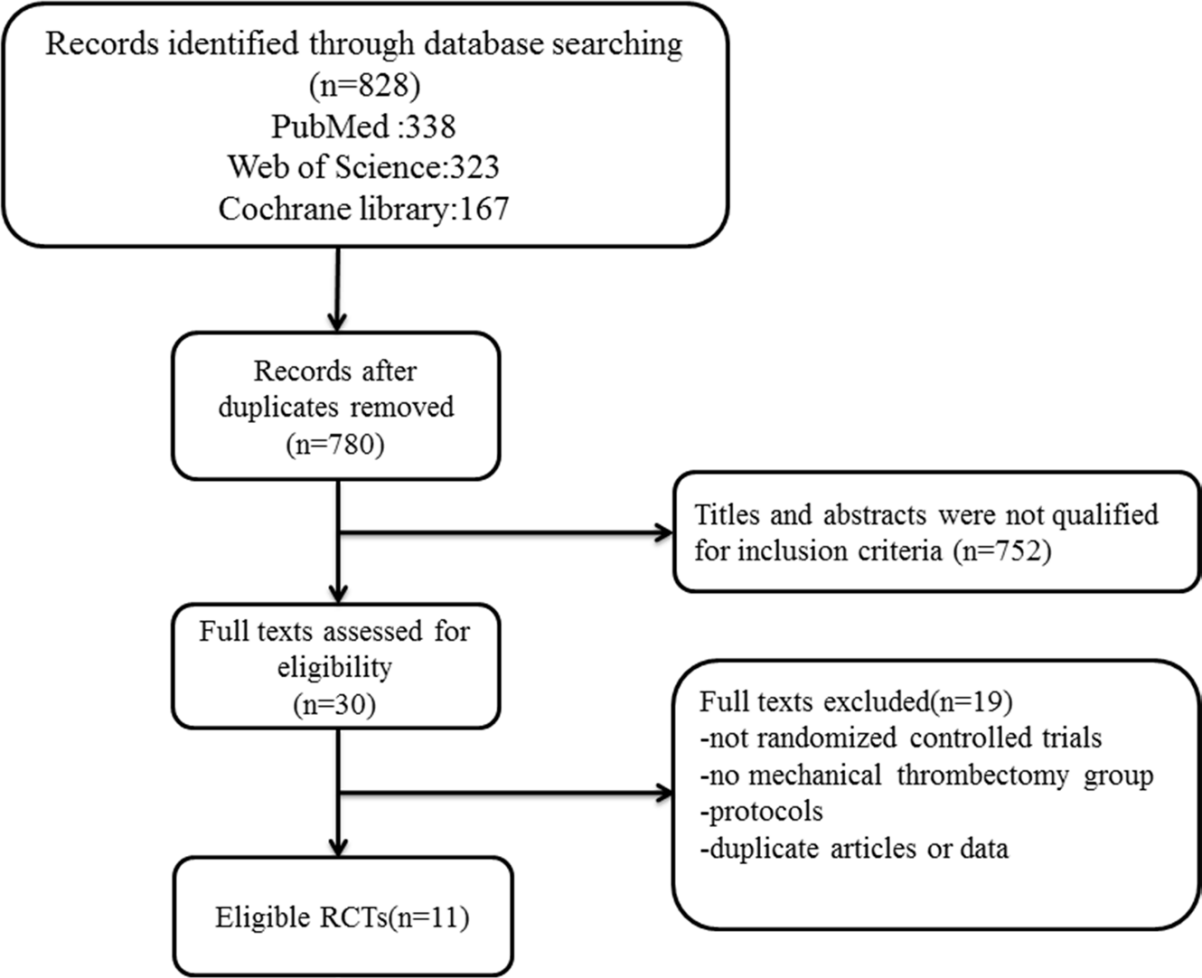
Search and selection process

This search strategy gathered 828 studies from PubMed, Web of Science, Cochrane Central Register of Controlled Trials (Figure 1). 752 studies were excluded after the screening because the titles and abstracts did not qualified. Then, 30 studies were assessed for eligibility, and 19 articles were excluded for the following reasons: 1 protocol, 3 not randomized controlled trials, 4 no mechanical thrombectomy group, 11 duplicate articles or data. The data were extracted from 11 RCTs: IMS III [14], MR RESCUE [15], SYNTHESIS [8], THRACE [13], EXTEND-IA [17], MR CLEAN [18], SWIFT PRIME [19], REVASCAT [20], ESCAPE [21], PISTE [22], THERAPY [16].

In these 11 studies (Table 1), the number of participants ranged from 65 to 656. All of these studies involved mechanical thrombectomy with t-PA as a part of the EVT, except the SYNTHESIS [8]. Patients in the EVT group were treated with different thrombectomy devices. In three RCTs (IMS III [14], MR RESCUE [15], SYNTHESIS [8]), patients were included without imaging confirmation of large vessel occlusion at the initial stage of the trials. The detailed data of study characteristics are shown in Table 1.

**Table 1:** Characteristics of studies included in the meta-analysis

Trials	No. of centers	Location	No. of patients	Devices	No. of IV t-PA(%)		Age	Time frame (h)		NIHSS	confirmation of large vessel occlusion
					t-PA	EVT		t-PA	EVT		
IMS III, 2013	58	North America, Europe, Australia	656	Merci Retriever, Penumbra System, Solitaire FR Device, Micro Sonic	100%	100%	18–82	3	5	≥ 10	NO*
MR RESCUE, 2013	22	North America	127	Merci Retriever, Penumbra System	29.6%	43.8%	18–85	4.5	8	29-Jun	NO*
SYNTHESIS, 2013	24	Italy	362	Solitaire FR Device, Penumbra System, Trevo device, Merci Retriever	96.1%	0	18–80	4.5	6	≤ 25	NO*
EXTEND-IA, 2015	14	Australia, New Zealand	70	Solitaire FR Device	100%	100%	≥ 18	4.5	6	__	YES
MR CLEAN, 2015	16	Netherlands	500	Solitaire FR Device, Merci retriever, Thromboaspiration, Wire disruption	90.6%	87.1%	≥ 18	4.5	6	≥2	YES
SWIFT PRIME, 2015	39	America, Europe	196	Solitaire FR or Solitaire 2	100%	100%	18–80	4.5	6	29-Aug	YES
REVASCAT, 2015	4	Spain	206	Solitaire FR	77.7%	68%	18–85	4.5	8	≥ 6	YES
ESCAPE, 2015	22	North America, Europe, South Korea	315	Solitaire FR,Thromboaspiration,	78.7%	72.7%	≥ 18	4.5	12	≥ 5	YES
THRACE, 2016	26	France	412	Solitaire, Penumbra, Trevo	100%	100%	18–80	4	5	25-Oct	YES
PISTE, 2017	10	United Kingdom	65	Stent-retriever, Aspiration	100%	100%	≥ 18	4.5	6	≥ 6	YES
THERAPY, 2016	36	US, German	108	Penumbra System, Solitaire FR, Trevo	100%	100%	18–85	3–4.5	8	≥ 8	YES

NIHSS: National Institutes of Health Stroke Scale; t-PA: intravenous thrombolysis with tissue plasminogen activator; EVT: endovascular treatment.

*At the beginning of the trials, patients were included without imaging confirmation of large vessel occlusion.

### Clinical outcomes

#### Primary outcomes

**Figure 2:**
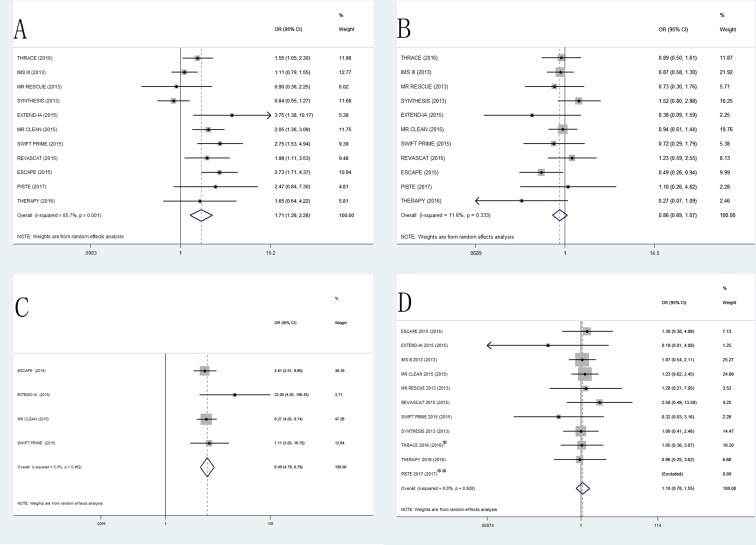
The pooled odd ratio of primary outcomes and secondary outcomes (**A**) mRS 0–2 at 90days, (**B**) all-cause mortality at 90 days, (**C**) recanalization rate at 24 h, (**D**) systematic intracranial hemorrhage (*: Systematic intracranial hemorrhage at 7 days in THRACE 2016. **: PISTE 2017 was excluded because that the incidence of sICH was zero).

In this meta-analysis, patients treated with EVT showed a higher proportion of favorable functional outcome at 90 days (OR, 1.71; 95% CI, 1.28–2.28; *P* < 0.001; Figure 2A) than those treated with standard medical therapy. There was moderate heterogeneity (*I*^2^ = 65.7%, *P* = 0.001) among the included RCTs.

### Secondary outcomes

Mortality at 90 days showed no significant difference in all-cause mortality at 90 days between EVT and standard medical therapy (OR, 0.86; 95% CI, 0.69–1.07; *P* = 0.165; Figure 2B). Four trials reported the revascularization rate at 24h in both EVT and standard medical therapy groups. EVT group had a better recanalization rate compared to the standard medical therapy group (OR, 6.49; 95% CI, 4.79–8.79; *P* < 0.001; Figure 2C). No significant difference was found in the incidence of sICH following EVT compared to standard medical therapy (OR, 1.10; 95% CI, 0.78–1.55; *P* = 0.584; Figure 2D).

### Meta regression and subgroup

**Figure 3:**
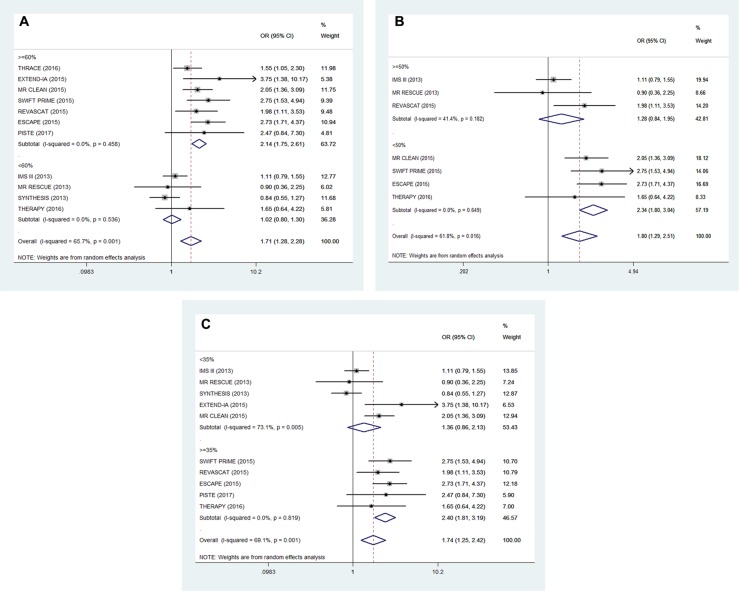
Subgroup analysis of primary outcomes (mRS 0–2 at 90 days) (**A**) low (< 60%) vs high (≥ 60%) rate of using Solitaire FR, (**B**) low (< 50%) and high (≥ 50%) percentage of patients with hyperlipidemia, (**C**) low (< 35%) vs high (≥ 35%) percentage of patients with atrial fibrillation.

**Figure 4:**
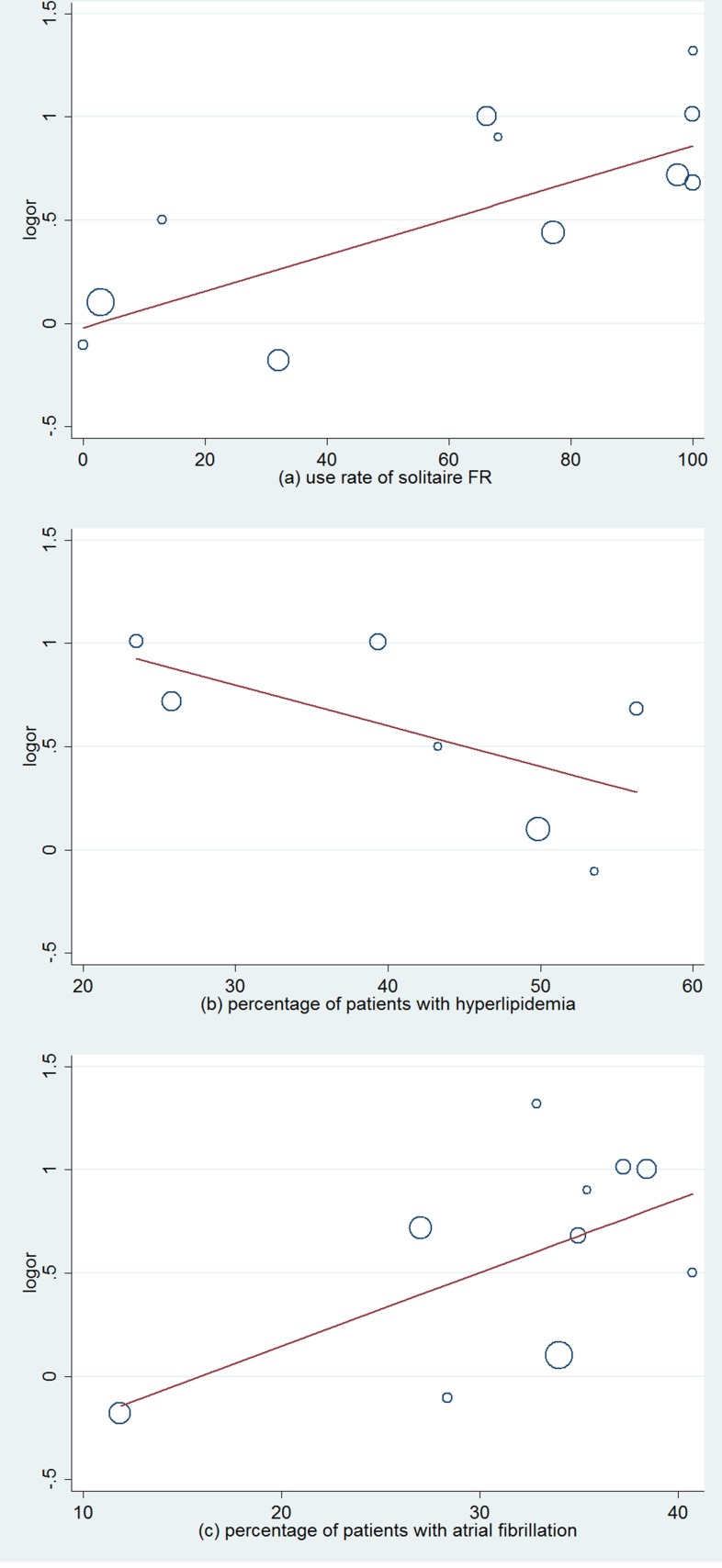
Meta-regression analysis of relative factors affecting the favorable functional outcomes

Moderate heterogeneity was observed among the 11 RCTs regarding favorable functional outcomes (*I*^2^ = 65.7%, *P* < 0.001). Hence, we conducted a univariate meta-regression regression analysis to find out the potential influencing factors. In the meta-regression, the rate of using solitaire FR could explain 72.21% of heterogeneity (*P* = 0.014, Adj R-squared = 72.21%; Table 2). In addition, hyperlipidemia and atrial fibrillation could respectively explain 35.21% (*P* = 0.159, Adj R-squared = 35.21%; Table 2) and 46.30% (*P* = 0.054, Adj R-squared = 46.30%; Table 2) of heterogeneity. In order to define the impact of these factors, we stratified the trials into low and high percentage of patients with corresponding characteristics (Figure 3 and Figure 4). In subgroup analysis, patients with the high use rate (> = 60%) of solitaire FR in EVT arm had favorable functional outcomes compared with standard medical care alone (OR, 2.14; 95% CI, 1.75–2.61; *P* < 0.001; Figure 3A). Meanwhile, in subgroups with the high percentage (> = 35%) of patients with atrial fibrillation, EVT also demonstrated an improving tendency in favorable functional outcomes (OR, 2.40; 95% CI, 1.81–3.19; *P* < 0.001; Figure 3C). Similar outcomes were also shown in subgroups with a low rate (< 50%) of patients with hyperlipidemia (OR, 2.34; 95% CI, 1.80–3.04; *P* < 0.001; Figure 3B).

**Table 2:** Single factor regression of the potential influence factors

Covariates	No. of RCTs	*P* value	Adj R-squared	coefficients	95%CI
Age	11	0.129	22.16%	0.089393	−0.03156 0.2103455
Male	11	0.315	6.89%	−3.61211	−11.29812 4.073904
Hypertension	11	0.58	−14.47%	−0.87801	−4.334825 2.578802
Diabetes	11	0.796	−12.49%	0.640172	−4.785959 6.066303
History of stroke	8	0.515	−199.06%	−2.25503	−10.23513 5.72507
Smoking	9	0.809	−84.45%	0.303401	−2.551318 3.158121
SBP	9	0.149	25.96%	−0.06661	−.1639032 0.0306782
blood glucose	8	0.879	−18.44%	−0.06317	−1.036518 0.9101708
NIHSS score	10	0.194	24.96%	0.136064	−0.0853451 0.3574724
Atrial fibrillation	10	0.054	46.30%	0.035621	−0.0008195 0.0720616
Antiplatelet therapy	6	0.336	23.80%	−2.83066	−10.02345 4.362135
Hyperlipidemia	7	0.159	35.21%	−0.01971	−0.0503131 0.0108966
IV t-PA in EVT group	11	0.069	31.45%	0.008095	−0.0007779 0.0169683
Use of solitaire FR	11	0.011	72.21%	0.008819	0.002606 0.0150324
ASPECT>7	8	0.081	58.50%	1.951249	−.3266271 4.229126
Site of occlusion					
ICA	9	0.197	100.00%	2.40161	−1.586371 6.389591
M1	9	0.309	100.00%	−1.40447	−4.429593 1.620653
M2	9	0.886	−102.77%	0.381309	−7.922869 8.369425
Time from onset to					
t-PA	10	0.122	33.04%	−0.00968	−0.0225864 0.0032263
Randomization	8	0.227	26.75%	0.008191	−0.0066995 0.0230816
groin puncture	7	0.631	−12.72%	0.004661	−0.0187709 0.028093
reperfusion	5	0.265	_	−0.00351	−0.0116583 0.0046468

NIHSS: National Institutes of Health Stroke Scale; SBP: systolic blood pressure; ICA: internal carotid artery; M1: M1 segment of Middle Cerebral Artery; M2: M2 segment of Middle Cerebral Artery; EVT: endovascular treatment t-PA: intravenous thrombolysis with tissue plasminogen activator; ASPECT: The Alberta Stroke Program Early Computed Tomography Score.

### Quality of the included studies

**Figure 5:**
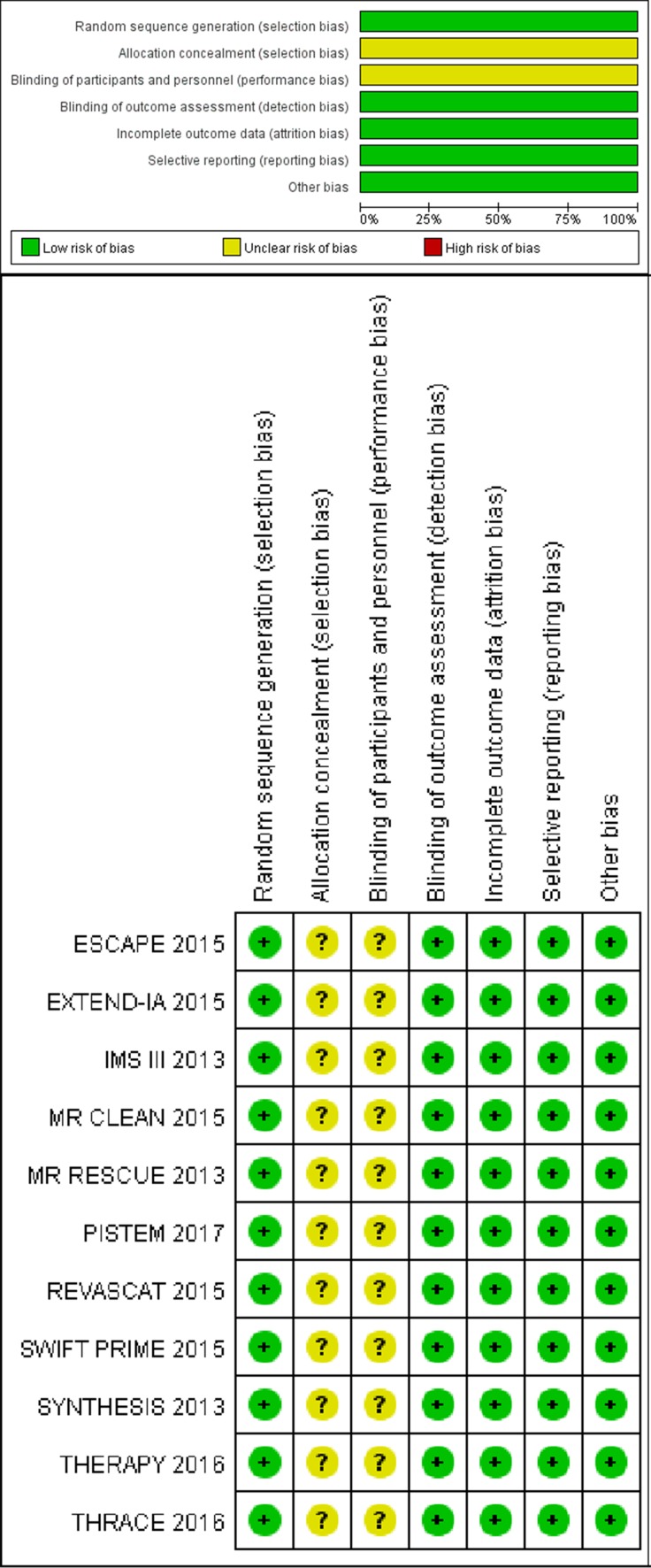
Risk of bias Review of the authors’ judgment about each risk of bias item for each included study based on the criteria recommended by the Cochrane Collaboration for randomized controlled trials.

**Figure 6:**
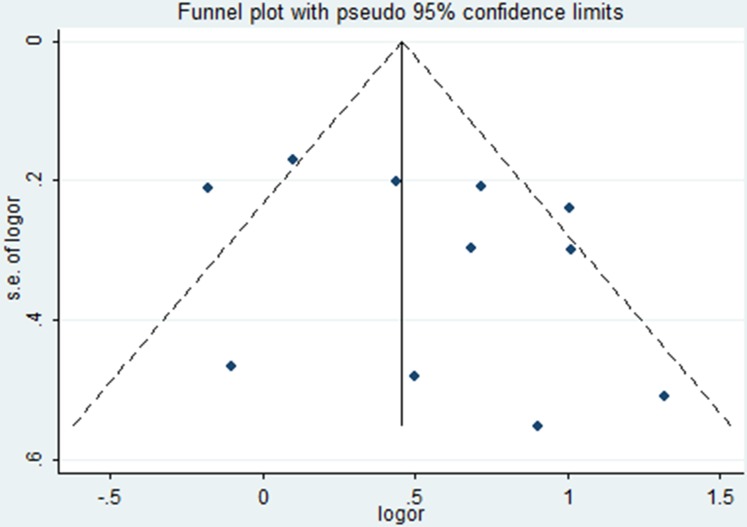
Funnel plot of included studies Funnel plot demonstrates possible evidence for publication bias in small studies, but larger studies show a symmetrical distribution of effects.

Publication bias was assessed by Egger's test (*P* = 0.256) and Begg's funnel plot analysis with pseudo 95% confidence limits (Figure 6), the result showed no risk. Concrete data about the risk of bias of each studies were shown in Figure 5. Though all studies were unblinding of participants and personnel to treatment allocation. We still consider the selection and performance bias as unclear risk of bias, since by study design blinding is impossible (PROBE design)

## DISCUSSION

This meta-analysis including 11 multicenter RCTs to evaluate the efficacy of EVT compared to standard medical therapy alone for patients with ischemic stroke. Our study indicated that EVT group was associated with high rates of favorable functional outcomeat 90 days and a favorable recanalization rate at 24 h when compared with medical care group. Nevertheless, no significant differences were observed in all-cause mortality at 90 days between EVT and standard medical care. Also, EVT was not associated with the high incidence rate of sICH at 90 days.

In our study, statistically considerable heterogeneity (*I*^2^ = 65.7%, *P* = 0.001) was found in favorable functional outcomes. Some factors could explain the discrepancy of different results between different RCTs. In the following RCTs, THERAPY 2016 [16], IMS III 2013 [14], MR RESCUE 2013 [15], and SYNTHESIS 2013 [8], the results showed similar safety outcomes and no significant difference in favorable functional outcomes with EVT, as compared with medical therapy alone. However, the subsequent RCTs, EXTEND-IA 2015 [17], MR CLEAN 2015 [18], SWIFT PRIME 2015 [19], REVASCAT 2015 [20], ESCAPE 2015 [21], THRACE 2016 [13], PISTE 2017 [22], were associated with high rates of functional independence. Such findings could be attributed to three considerable flaws of 4 former RCTs. In these RCTs (IMS III 2013, MR RESCUE 2013, SYNTHESIS 2013 and THERAPY 2016), intra-arterial t-PA or first-generation mechanical thrombectomy devices played a primary role in EVT. Such treatment may result in poor efficiency and related complications of EVT [11, 25]. Similarly, because of the different inclusion criteria, these trials selected patients without imaging evidence of large vessel occlusion, which would lead to the enrollment of patients with distal or no artery occlusion. Moreover, irregular use of IV t-PA in EVT group, especially in SYNTHESIS 2013 (0%) and MR RESCUE (43.8%), may be another non-negligible factor of the heterogeneity in our meta-analysis. The subsequent trials (EXTENT-IA 2015, MR CLEAN 2015, SWIFT PRIME 2015, REVASCAT 2015, ESCAPE 2015, THRACE 2016, PISTE 2017) ameliorated these limitations. In these recent 7 trials, patients were included with the confirmation of large vessel occlusion, and the rate of use of IV t-PA was high (at least 77.7% in IVT group, 68% in EVT group). The rate of employing thrombectomy devices in EVT group was up to at least 68% in trials after 2015 (Supplementary Table 1). Meanwhile, these trials used the newer generation device solitaire FR in > 60% of cases in patients with thrombectomy (Supplementary Table 2). The solitaire FR Device achieved higher recanalization rates based on angiographic evidence, and also better neurological outcomes when compared with the Merci Retrieval System [11], the first proven device approved by FDA.

Actually, the above-mentioned 3 factors have been detailedly investigated in recent meta-analysis [4, 23, 24]. Hence, we attempted to implement the univariate meta-regression to identify the potential factors which were missed previously (Because of the limitation of numbers of RCTs, we chose univariate meta-regression instead of multi-factor regression). In our research, the univariate meta-regression indicated that the rate of using solitaire FR (*P* = 0.011, Adj R-squared = 72.21%) was associated with the heterogeneity of our study. Subgroup analysis indicated that patients with high use rate (>= 60%) of solitaire FR in the EVT group had favorable functional outcomes compared with standard medical care alone (OR, 2.14; 95% CI, 1.75–2.61; *P* < 0.001; Figure 3A). Such results further confirmed the influence of the significant flaws in 2013 RCTs, and suggested that the rate of using solitaire FR was the predominant influence factor behind the heterogeneity. Furthermore, two newfound factors, hyperlipidemia and atrial fibrillation, caught our attention (Figure 4B–4C). These two factors had never been reported in recent meta-analysis [4, 23, 24]. In the subgroup with a high percentage (>= 35%) of stroke patients with atrial fibrillation, EVT showed an increased effectiveness (OR, 2.40; 95% CI, 1.81–3.19; P < 0.001; Figure 3C). On the contrary, hyperlipidemia showed a negative correlation with the favorable functional outcomes (Figure 4B). In the subgroup with low percentage (< 50%) of patients with hyperlipidemia, EVT also demonstrated an improving tendency in favorable functional outcomes (OR, 2.34; 95% CI, 1.80–3.04; P < 0.001; Figure 3B). This phenomenon may have some plausible explanations.

Stroke of arterial origin can arise from cardioembolism, large artery atherosclerosis and small vessel disease (SVD) [26]. Hyperlipidemia is one of the major risk factors of stroke [27], and is thought to be strongly related to the diagnosis of SVD [28]. SVD is the most frequent pathological processes affecting small vessels and capillaries of the brain and play a crucial role in stroke. As an expression of SVD, lacunar stroke is associated with an excess risk of disability and death [29]. Apparently, patients with SVD can hardly obtain benefits from mechanical thrombectomy. However, IV t-PA is still a useful treatment for lacunar stroke, like the other stroke subtypes [30]. These reasons lead to the significant difference in the subgroup analysis which stratified the trials into low (< 50%) and high (>= 50%) percentage of patients with hyperlipidemia (Figure 3). Meanwhile, in subgroup with high rate (>= 50%) of patients with hyperlipidemia, two of these 3 RCTs selected patients without imaging evidence of large vessel occlusion at the beginning of the trials (Figure 3, Table 1). It likely leads to the enrollment of patients with SVD.

In subgroup with a high percentage (>= 35%) of patients with atrial fibrillation, EVT was associated with increased effectiveness (Figure 3C, Figure 4C). Indeed, atrial fibrillation is the crucial cause of cardioembolic stroke (CES) which accounts for 1/5 incidence of ischemic stroke [31, 32], Meanwhile, atrial fibrillation-related (AF-related) stroke is thought to be linked to a worse 90-days outcomes when compared with other CES subtypes [32]. Ischemic stroke patients with AF can hardly benefit from intravenous thrombolysis when compare with non-AF patients [33, 34]. Hence, we speculated that the embolus type may be a potential influence factor affecting the recanalization rate and functional outcomes. Patients with AF-related CES can hardly gain benefits from IV t-PA [32]. But they can benefit from mechanical thrombectomy. This speculation is consistent with our analysis results (Figures 3, 4). A recent study suggested that mechanical thrombectomy can significantly improve the short- and long-term clinical outcomes of patients with cardioembolic stroke when compared with IV t-PA [35]. This result further corroborated our conjecture.

### Limitation

There are numbers of limitations in our meta-analysis. Although mechanical thrombectomy plays an important role in the treatment of EVT, we can't ignore the influence of chemical thrombolytic treatment such as intra-arterial t-PA. Furthermore, we didn't include some factors into the univariate meta-regression because of insufficient data (imaging location, time from onset to imaging, congestive heart failure, coronary artery disease and so on). These missing data may lead to the neglect of other potential important factors. Moreover, due to the limited data, we could only conduct a simple stratified analysis to study the impact of hyperlipidemia and atrial fibrillation. A quantitative analysis should be done to determine the influence of different levels of blood lipids. Likewise, diverse types of atrial fibrillation are related to different long-term outcomes in patients with stroke [36]. Hence, it's of value to explore the individual treatment to ischemic stroke patients with diverse types of atrial fibrillation.

## MATERIALS AND METHODS

This meta-analysis was reported following the *Preferred Reporting Items for Systematic Reviews and Meta-Analyses* (PRISMA) guidelines [37].

### Search method

The investigators searched for studies published between January 2011 and April 2017 using several databases (PubMed, web of science, Cochrane Central Register of Controlled Trials). The search terms included “mechanical thrombolysis”, “thrombectomy”, “mechanical thrombectomy”, “endovascular therapy”, “embolectomy”, “endovascular treatment”, “endovascular thrombectomy” AND “Stroke”, “acute ischemic stroke”, “ischemic stroke”, “cerebrovascular accident”, AND “randomized controlled trial”. The references of eligible articles were also selected in case of the omissions of RCTs.

### Eligibility criteria

RCTs comparing EVT (t-PA with adjunctive embolectomy, mechanical thrombectomy alone or intra-arterial thrombolysis) with standard medical care alone were included in our study. Meanwhile, these eligible studies had to report the mRS and mortality at 90 days of patients >18 years with AIS. In addition, mechanical thrombectomy must be part of the EVT and patients should be treated with intravenous t-PA in control group. Moreover, we excluded RCTs in which the number of participants was < 20 or the follow-up was less than 90 day.

### Selection of studies

Two reviewers (Z.J.W. and Z.N.N.) independently screened the titles and abstracts of the studies. Then the full texts would be assessed if the article passed the initial screening. Disagreements were solved by discussion or with the help of two other reviewers (S.L.G. and X.W.L.)

### Data extraction

Two reviewers (S.L.G. and Z.J.W.) independently extracted data from the eligible studies and supplementary materials. We used a standardized data extraction form to collect the following data: Characteristics of eligible studies (trial name, regions, number of centers, phases, publications, inclusion criteria, exclusion criteria, study design, efficacy outcome, safety outcome); Characteristics of patients (population, sex ratio, National Institutes of Health Stroke Scale (NIHSS), Alberta Stroke Program Early Computed Tomography Score (ASPECTS), time from onset to imaging, time from onset to intravenous thrombolysis, time from onset to randomization, time from onset to thrombectomy, Occlusion site, et al.). Two reviewers (X.W.L. and L.F.) independently evaluated any controversial data, and reconfirmed the extracted data.

### Outcome measures and quality assessment

The primary outcome was the number of patients with favorable functional outcomes (mRS 0–2) at 90 days. The secondary outcomes included recanalization rate at 24 h, all-cause mortality at 90 days and incidence rate of sICH at 90 days. In addition, we also evaluated whether the types of thrombectomy devices or the characteristics of patients (atrial fibrillation, hyperlipidemia) would affect the clinical outcomes leading to heterogeneity. According to the results of univariate meta-regression, we set the relevant cut-off values that could distinctly affect the effectiveness of EVT or IV t-pA (Figure 4), and the heterogeneity may also be decreased in the subsequent subgroup analysis [38, 39]. Thus, we stratified the included trials into low (< 50%) and high (≥ 50%) percentage of patients with hyperlipidemia, low (< 35%) vs high (≥ 35%) percentage of patients with atrial fibrillation and low (< 60%) vs high (≥ 60%) rate of using Solitaire FR (Figure 3).

Biases of the included RCTs were assessed independently by 2 investigators (Z.J.W. and S.L.G.) using the Cochrane Collaboration risk of bias tool [28]. The items contained selection bias, performance bias, detection bias, attrition bias, reporting bias, and other potential biases. Each items was categorized as high, low, or unclear risks.

### Statistical analysis

In our study, all outcomes were conducted by using the STATA 12 with a random effect model because of the possible heterogeneity. The heterogeneity among studies was measured by *I*^2^ value (low: < 25%, moderate: 25%-70%, high: > 70%) [40]. Univariate meta-regression (mixed-effects restricted maximum likelihood models) was used to find out the potential factors which could account for the heterogeneity. We collected the summary data of baseline patient characteristics (Supplementary Table 3) without selective elimination, and then deleted the variables which were reported in less than 4 RCTs (insufficient data may lead to the invalidity of meta-regression). The remaining variables which act as the covariates would participate in the process of meta-regression. Adjusted R square was the crucial index for us to find the heterogeneity, it represented the proportion of between-study variance explained by the covariates [41]. Variables with ideal Adjusted R square would get further processing. Subgroup analysis was used to further confirm the influence of these factors. The primary and secondary outcomes were measured by odds ratios (ORs) with corresponding 95% confidence intervals. The Cochrane collaboration's tool was used to estimate the risk of bias with RCTs. Meanwhile, Begg's funnel plot analysis was used to assess for publication bias.

## CONCLUSIONS

EVT was associated with favorable functional outcomes and higher recanalization rate with imaging evidence compared with standard medical care alone. The incidence of symptomatic intracranial hemorrhage and mortality were similar among patients with EVT or IVT. The rate of using the new generation thrombectomy device was the most important factor influencing the effectiveness of EVT. Furthermore, hyperlipidemia and atrial fibrillation may be potential factors affecting therapy outcomes.

## SUPPLEMENTARY MATERIALS TABLES



## References

[1] Hankey GJ (2017). Stroke. Lancet.

[2] Thrift AG, Thayabaranathan T, Howard G, Howard VJ, Rothwell PM, Feigin VL, Norrving B, Donnan GA, Cadilhac DA (2017). Global stroke statistics. Int J Stroke.

[3] Warach S, Johnston SC (2016). Endovascular Thrombectomy for Ischemic Stroke: The Second Quantum Leap in Stroke Systems of Care?. JAMA.

[4] Badhiwala JH, Nassiri F, Alhazzani W, Selim MH, Farrokhyar F, Spears J, Kulkarni AV, Singh S, Alqahtani A, Rochwerg B, Alshahrani M, Murty NK, Alhazzani A (2015). Endovascular Thrombectomy for Acute Ischemic Stroke: A Meta-analysis. JAMA.

[5] Leiva-Salinas C, Patrie JT, Xin W, Michel P, Jovin T, Wintermark M (2016). Prediction of Early Arterial Recanalization and Tissue Fate in the Selection of Patients With the Greatest Potential to Benefit From Intravenous Tissue-Type Plasminogen Activator. Stroke.

[6] de Los Rios la Rosa F, Khoury J, Kissela BM, Flaherty ML, Alwell K, Moomaw CJ, Khatri P, Adeoye O, Woo D, Ferioli S, Kleindorfer DO (2012). Eligibility for Intravenous Recombinant Tissue-Type Plasminogen Activator Within a Population: The Effect of the European Cooperative Acute Stroke Study (ECASS) III Trial. Stroke.

[7] Jauch EC, Saver JL, Adams HP, Bruno A, Connors JJ, Demaerschalk BM, Khatri P, McMullan PW, Qureshi AI, Rosenfield K, Scott PA, Summers DR, Wang DZ (2013). Guidelines for the early management of patients with acute ischemic stroke: a guideline for healthcare professionals from the American Heart Association/American Stroke Association. Stroke.

[8] Ciccone A, Valvassori L, Nichelatti M, Sgoifo A, Ponzio M, Sterzi R, Boccardi E, Investigators SE (2013). Endovascular treatment for acute ischemic stroke. N Engl J Med.

[9] Pierot L, Soize S, Benaissa A, Wakhloo AK (2015). Techniques for endovascular treatment of acute ischemic stroke: from intra-arterial fibrinolytics to stent-retrievers. Stroke.

[10] Meyers PM, Schumacher HC, Connolly ES, Heyer EJ, Gray WA, Higashida RT (2011). Current status of endovascular stroke treatment. Circulation.

[11] Saver JL, Jahan R, Levy EI, Jovin TG, Baxter B, Nogueira RG, Clark W, Budzik R, Zaidat OO, Trialists S (2012). Solitaire flow restoration device versus the Merci Retriever in patients with acute ischaemic stroke (SWIFT): a randomised, parallel-group, non-inferiority trial. Lancet.

[12] Kharitonova TV, Melo TP, Andersen G, Egido JA, Castillo J, Wahlgren N, investigators S (2013). Importance of cerebral artery recanalization in patients with stroke with and without neurological improvement after intravenous thrombolysis. Stroke.

[13] Bracard S, Ducrocq X, Mas JL, Soudant M, Oppenheim C, Moulin T, Guillemin F (2016). Mechanical thrombectomy after intravenous alteplase versus alteplase alone after stroke (THRACE): a randomised controlled trial. Lancet Neurol.

[14] Broderick JP, Palesch YY, Demchuk AM, Yeatts SD, Khatri P, Hill MD, Jauch EC, Jovin TG, Yan B, Silver FL, von Kummer R, Molina CA, Demaerschalk BM (2013). Endovascular therapy after intravenous t-PA versus t-PA alone for stroke. N Engl J Med.

[15] Kidwell CS, Jahan R, Gornbein J, Alger JR, Nenov V, Ajani Z, Feng L, Meyer BC, Olson S, Schwamm LH, Yoo AJ, Marshall RS, Meyers PM (2013). A trial of imaging selection and endovascular treatment for ischemic stroke. N Engl J Med.

[16] Mocco J, Zaidat OO, von Kummer R, Yoo AJ, Gupta R, Lopes D, Frei D, Shownkeen H, Budzik R, Ajani ZA, Grossman A, Altschul D, McDougall C (2016). Aspiration Thrombectomy After Intravenous Alteplase Versus Intravenous Alteplase Alone. Stroke.

[17] Campbell BC, Mitchell PJ, Kleinig TJ, Dewey HM, Churilov L, Yassi N, Yan B, Dowling RJ, Parsons MW, Oxley TJ, Wu TY, Brooks M, Simpson MA (2015). Endovascular therapy for ischemic stroke with perfusion-imaging selection. N Engl J Med.

[18] Berkhemer OA, Fransen PS, Beumer D, van den Berg LA, Lingsma HF, Yoo AJ, Schonewille WJ, Vos JA, Nederkoorn PJ, Wermer MJ, van Walderveen MA, Staals J, Hofmeijer J (2015). A randomized trial of intraarterial treatment for acute ischemic stroke. N Engl J Med.

[19] Saver JL, Goyal M, Bonafe A, Diener HC, Levy EI, Pereira VM, Albers GW, Cognard C, Cohen DJ, Hacke W, Jansen O, Jovin TG, Mattle HP (2015). Stent-retriever thrombectomy after intravenous t-PA vs. t-PA alone in stroke. N Engl J Med.

[20] Jovin TG, Chamorro A, Cobo E, de Miquel MA, Molina CA, Rovira A, San Roman L, Serena J, Abilleira S, Ribo M, Millan M, Urra X, Cardona P (2015). Thrombectomy within 8 hours after symptom onset in ischemic stroke. N Engl J Med.

[21] Goyal M, Demchuk AM, Menon BK, Eesa M, Rempel JL, Thornton J, Roy D, Jovin TG, Willinsky RA, Sapkota BL, Dowlatshahi D, Frei DF, Kamal NR (2015). Randomized assessment of rapid endovascular treatment of ischemic stroke. N Engl J Med.

[22] Muir KW, Ford GA, Messow CM, Ford I, Murray A, Clifton A, Brown MM, Madigan J, Lenthall R, Robertson F, Dixit A, Cloud GC, Wardlaw J (2017). Endovascular therapy for acute ischaemic stroke: the Pragmatic Ischaemic Stroke Thrombectomy Evaluation (PISTE) randomised, controlled trial. J Neurol Neurosurg Psychiatry.

[23] Balami JS, Sutherland BA, Edmunds LD, Grunwald IQ, Neuhaus AA, Hadley G, Karbalai H, Metcalf KA, DeLuca GC, Buchan AM (2015). A systematic review and meta-analysis of randomized controlled trials of endovascular thrombectomy compared with best medical treatment for acute ischemic stroke. Int J Stroke.

[24] Bush CK, Kurimella D, Cross LJ, Conner KR, Martin-Schild S, He J, Li C, Chen J, Kelly T (2016). Endovascular Treatment with Stent-Retriever Devices for Acute Ischemic Stroke: A Meta-Analysis of Randomized Controlled Trials. PLoS One.

[25] Mullen MT, Pisapia JM, Tilwa S, Messe SR, Stein SC (2012). Systematic Review of Outcome After Ischemic Stroke Due to Anterior Circulation Occlusion Treated With Intravenous, Intra-Arterial, or Combined Intravenous plus Intra-Arterial Thrombolysis. Stroke.

[26] Niesten JM, van der Schaaf IC, Biessels GJ, van Otterloo AE, van Seeters T, Horsch AD, Luitse MJ, van der Graaf Y, Kappelle LJ, Mali WP, Velthuis BK (2013). Trial DUaS. Relationship between thrombus attenuation and different stroke subtypes. Neuroradiology.

[27] Shigematsu K, Watanabe Y, Nakano H, Kyoto Stroke Registry C (2015). Influences of hyperlipidemia history on stroke outcome; a retrospective cohort study based on the Kyoto Stroke Registry. BMC Neurol.

[28] Ihle-Hansen H, Thommessen B, Wyller TB, Engedal K, Fure B (2012). Risk factors for and incidence of subtypes of ischemic stroke. Funct Neurol.

[29] Norrving B (2003). Long-term prognosis after lacunar infarction. Lancet Neurol.

[30] Pantoni L, Fierini F, Poggesi A (2014). Thrombolysis in acute stroke patients with cerebral small vessel disease. Cerebrovasc Dis.

[31] Higashida RT, Furlan AJ, Roberts H, Tomsick T, Connors B, Barr J, Dillon W, Warach S, Broderick J, Tilley B, Sacks D (2003). Technology Assessment Committee of the American Society of Interventional and Therapeutic Neuroradiology; Technology Assessment Committee of the Society of Interventional Radiology. Trial design and reporting standards for intra-arterial cerebral thrombolysis for acute ischemic stroke. Stroke.

[32] Henninger N, Goddeau RP, Karmarkar A, Helenius J, McManus DD (2016). Atrial Fibrillation Is Associated With a Worse 90-Day Outcome Than Other Cardioembolic Stroke Subtypes. Stroke.

[33] Kimura K, Iguchi Y, Shibazaki K, Iwanaga T, Yamashita S, Aoki J (2009). IV t-PA therapy in acute stroke patients with atrial fibrillation. J Neurol Sci.

[34] Zhang JB, Ding ZY, Yang Y, Sun W, Hai F, Sui XN, Li XY, Wang HZ, Wang XT, Zheng JL (2010). Thrombolysis with alteplase for acute ischemic stroke patients with atrial fibrillation. Neurol Res.

[35] Fu M, He W, Dai W, Ye Y, Ruan Z, Wang S, Xie H (2016). Efficacy of Solitaire™ Stent Arterial Embolectomy in Treating Acute Cardiogenic Cerebral Embolism in 17 Patients. Med Sci Monitor.

[36] Ntaios G, Vemmou A, Koroboki E, Savvari P, Makaritsis K, Saliaris M, Andrikopoulos G, Vemmos K (2013). The type of atrial fibrillation is associated with long-term outcome in patients with acute ischemic stroke. Int J Cardiol.

[37] Moher D, Liberati A, Tetzlaff J, Altman DG (2009). Preferred reporting items for systematic reviews and meta-analyses: the PRISMA statement. Ann Intern Med.

[38] Shi XQ, Wang ZZ (2008). [Application of Meta-regression and subgroup analyses of heterogeneity disposal in Meta-analysis]. [Article in Chinese]. Chin J Epidemiol.

[39] Thompson SG, Higgins JPT (2002). How should meta-regression analyses be undertaken and interpreted?. Stat Med.

[40] Higgins JPT, Altman DG, Gotzsche PC, Juni P, Moher D, Oxman AD, Savovic J, Schulz KF, Weeks L, Sterne JAC (2011). Cochrane Bias Methods Group; CochraneStatistical Methods Group. The Cochrane Collaboration's tool for assessing risk of bias in randomised trials. BMJ.

[41] Harbord RM, Higgins JPT (2008). Meta-regression in Stata. Stata J.

