# Supplementary material for: Impact of hyperlipidemia and atrial fibrillation on the efficacy of endovascular treatment for acute ischemic stroke: a meta-analysis
Source: Oncotarget. 2017 Aug 11;8(42):72972–84. doi: 10.18632/oncotarget.20183 (PMC5641184; doi:10.18632/oncotarget.20183)
Supplement: Supplementary file 1 [file oncotarget-08-72972-s001.pdf]

Impact of hyperlipidemia and atrial fibrillation on the efficacy of endovascular treatment for acute ischemic stroke: a meta-analysis

SUPPLEMENTARY MATERIALS

Supplementary Table 1: No. of patients with mechanical thrombectomy

Trials	No. of patients with endovascular treatment	No. of patients with thrombectomy device (%)
IMS III 2013	434	170 (39)
MR RESCUE 2013	64	61 (95)
SYNTHESIS 2013	181	56 (31)
EXTEND-IA 2015	35	27 (77)
MR CLEAN 2015	233	190 (82)
SWIFT PRIME 2015	98	87 (89)
REVASCAT 2015	103	98 (95)
ESCAPE 2015	165	130 (79)
THRACE 2016	204	140 (68)
PISTEM 2017	33	30 (90)
THERAPY 2016	55	45 (82)

Supplementary Table 2: Use rate of solitaire FR

trials	Use rate of Solitaire FR (%)
**IMS III 2013**	2.9%
**MR RESCUE 2013**	0%
**SYNTHESIS 2013**	32.1%
**EXTEND-IA 2015**	100%
**MR CLEAN 2015**	97.5%
**SWIFT PRIME 2015**	100%
**REVASCAT 2015**	100%
**ESCAPE 2015**	66.2%
**THRACE 2016**	77.1%
**PISTEM 2017**	68%
**THERAPY 2016**	13%

Supplementary Table 3: Baseline characteristics of patients in included randomized controlled trials.
See Supplementary_Table_3
